# Structural and morphological tuning of Cu-based metal oxide nanoparticles by a facile chemical method and highly electrochemical sensing of sulphite

**DOI:** 10.1038/s41598-021-82741-z

**Published:** 2021-02-09

**Authors:** Velayutham Sudha, Govindhasamy Murugadoss, Rangasamy Thangamuthu

**Affiliations:** 1 Electroorganic and Materials Electrochemistry (EME) Division, CSIR-Central Electrochemical Research Institute (CSIR-CECRI), Karaikudi, Tamil Nadu 630 003 India; 2 Academy of Scientific and Innovative Research (AcSIR), Ghaziabad, 201 002 India; 3 Centre for Nanoscience and Nanotechnology, Sathyabama Institute of Science and Technology, Chennai, Tamil Nadu 600 119 India

**Keywords:** Nanoscale materials, Electrochemistry

## Abstract

A facile one-step chemical method is introduced for the successful synthesis of Cu_2_O, CuO and CuNa_2_(OH)_4_ crystal structures and their electrochemical properties were also investigated. X-ray diffraction studies revealed that these copper-based oxide nanoparticles display different crystal structures such as cubic (Cu_2_O), monoclinic (CuO) and orthorhombic [CuNa_2_(OH)_4_]. The microstructural information of nanoparticles was investigated by transmission electron microscopy. It shows attractive morphologies of different orientation such as rod like structure, nanobeads and well-aligned uniform nanorod for Cu_2_O, CuO and CuNa_2_(OH)_4_, respectively. Electrochemical sensing of sulphite (SO_3_^2−^) on these three copper-based oxide modified electrodes was investigated. Among the three different crystal structures, CuO shows promising electrocatalytic activity towards oxidation of sulphite. A linear variation in peak current was obtained for SO_3_^2−^ oxidation from 0.2 to 15 mM under the optimum experimental condition. The sensitivity and detection limit were in the order of 48.5 µA cm^−2^ mM^−1^ and 1.8 µM, respectively. Finally, practical utility of CuO modified electrode was demonstrated for the estimation of sulphite in commercial wine samples.

## Introduction

Electrochemical sensor research is one of the important areas because of its application in numerous fields including drug, industrial, food, environmental and so on. Sulphite (SO_3_^2−^) is used as a food additive and also as an inhibitor to prevent the microbial reactions^1^. Sulphite improves the appearance of foods and wines and maintains their quality as well. The US-FDA recommended levels of SO_3_^2−^ in food are below 10 mg/kg and liquid items 10 mg/L^-^^1^^2^. Excess amount of SO_3_^2−^ in food products leads to form number of symptoms which includes asthma in our human body and number of changes in the organoleptic properties of raw materials. In some cases, till now SO_3_^2−^ was used in wine and some other food as an additive because other additives are not found for the replacement of SO_3_^2−^. On the other hand, SO_3_^2−^ is a precursor to produce acid rain, which acidifies the water bodies, soil and harms trees, crops, monuments and buildings^3,4^. Therefore, estimation of SO_3_^2−^ is significant in the trace analysis of food, surface water and drinking water and so on.

The Association of Analytical Chemists (AOAC) has recommended Monier-Williams method as a standard method for the detection of sulphite. Besides long analysis time, the conventional titrimetric method also suffers from poor precision. In the pursuit of suitable alternative, a number of methods have been established for the detection of SO_3_^2−^ such as ion chromatography^5^, FIA/gas diffusion^6^, spectrophotometric detection^7–12^, flow injection analysis (FIA)^13^, colorimetric titration^14^, chemiluminescence^15,16^, and electrochemical estimation^17–19^. Among these methods, electrochemical technique is attractive due to high selectivity, sensitivity, wide concentration range, low-cost, simplicity and so on. The electrooxidation of SO_3_^2−^ on conventional electrodes shows high over potential due to sluggish electron transfer^20,21^. Therefore, the electrochemical method based on modified electrodes is an attractive approach for the detection of SO_3_^2−^.

Nanomaterials accomplish an essential part in the electrochemical sensing of trace amount food additives, pharmaceutical compounds, harmful pollutants and heavy metal ions as nanomaterials show different physico-chemical properties compared with bulk one. Novel electrochemical sensors based on nanostructured catalysts were developed^22–24^. Nowadays, sensing platform based on nanostructured metal oxides especially copper oxide, CuO nanocomposites with other transition metal oxides and carbon materials were explored for determination of different analytes. CuO is a P-type semiconductor with narrow bandgap (E_g_) of 1.2 eV, which shows attractive properties such as high electrical conductivity, good stability, efficient electrode in photovoltaics, high mechanical strength, high catalytic activity and high temperature durability. Moreover, CuO is low cost and abundant non-toxic semiconductor^25–29^. The CuO based materials are widely used in gas sensor, photocatalyst and lithium ion electrode materials. Recently, a number of electroactive sensor platforms were extensively used to analyse pharmaceutical and biologically important compounds^30–34^. Numerous methods including sol–gel, hydrothermal, sonochemical, thermal evaporation, microwave irradiation and electrochemical approach have been reported for preparation of CuO, Cu_2_O and other coper oxide-based nanomaterials. Even though some of the above methods seem as simple, but it is hard to control crystal structures and morphology using a single method. Therefore, it is essential to design and development of a unique synthesis method for preparation of different crystal structures with different morphology by the single method in industrial-scale. In this direction, for the first time we have prepared different types of Cu based nanomaterials such as CuNa_2_(OH)_4_, Cu_2_O and CuO by a facile one-step chemical method using CuSCN as source material. The synthesized materials were characterized using several advanced techniques and their electrocatalytic activity was evaluated towards oxidation of SO_3_^2−^. Practical application of CuO modified electrode was successfully demonstrated for the determination of SO_3_^2−^ in wine samples.

## Experimental section

### Materials and reagents

Copper (I) thiocynate (CuSCN, 99%) was purchased from Aldrich. Sodium hydroxide (98%), polyvinylpyrrolidone (PVP; WM=40000) and hydrazine hydrate (≥ 80%) were purchased from Loba Chemie, India. Monosodium dihydrogen phosphate (NaH_2_PO_4_, ≥ 99%) and disodium hydrogen phosphate (Na_2_HPO_4_, ≥ 99%) were used to prepare 0.1 M phosphate buffer solution (PBS; pH 7). For the whole experimental work, ultrapure water (18.2 MΩ) was used.

### Synthesis of the copper-based metal oxides

To synthesis various structure of copper-based oxide nanoparticles, a simple solution phase method was used. Typically, 0.2 M copper (I) thiocyanate (CuSCN) was dissolved in de-ionized water. Next, 1 g of PVP was added into the above solution and stirred until completely dissolved. Then, 0.25, 0.5, 1.0 M NaOH pellets were dropped into the above solution followed by addition of constant volume of 5 ml hydrazine hydrate. The resultant solution was stirred for 2 h at room temperature. Then the obtained precipitate was washed by repeated centrifugation at 4000 rpm for 20 min. Finally, the wet samples were dried at 120 °C for 6 h.

### Materials and electrochemical characterizations

Characterization of Cu based metal oxide nanoparticles were carried out by using following techniques. The morphological study and energy dispersive X-ray analysis (EDX) were performed using scanning electron microscopy (SEM) by SEM-JEOL JSM-6380LV. X-ray diffraction pattern (XRD) of the powder samples was obtained with PW3040/60 X’pert PRD X-ray powder diffractometer equipped with a scintillation counter using Cu Kα radiation (λ = 0.1540 nm). The transmission electron microscopy (TEM) characterization was carried out using JEM 2100 F with 200 kV acceleration voltages. ESCA+Omicron UK XPS system was used for X-ray photoelectron spectroscopy (XPS) analysis with an Mg-Kα source. The functional group analysis was performed with Thermo Nicolet 200. In the electrochemical studies we used three electrode system and AUTOLAB PGSTAT302N (NOVA) instrument. Working electrodes are CuO/GCE, Cu_2_O/GCE and CuNa_2_(OH)_4_/GCE, reference electrode is saturated calomel electrode and counter electrode is platinum wire. In order to remove the dissolved oxygen, the experimental solution was purged with high purity inert N_2_ gas.

## Results and discussion

### Physical characterization of the samples

**Figure 1:**
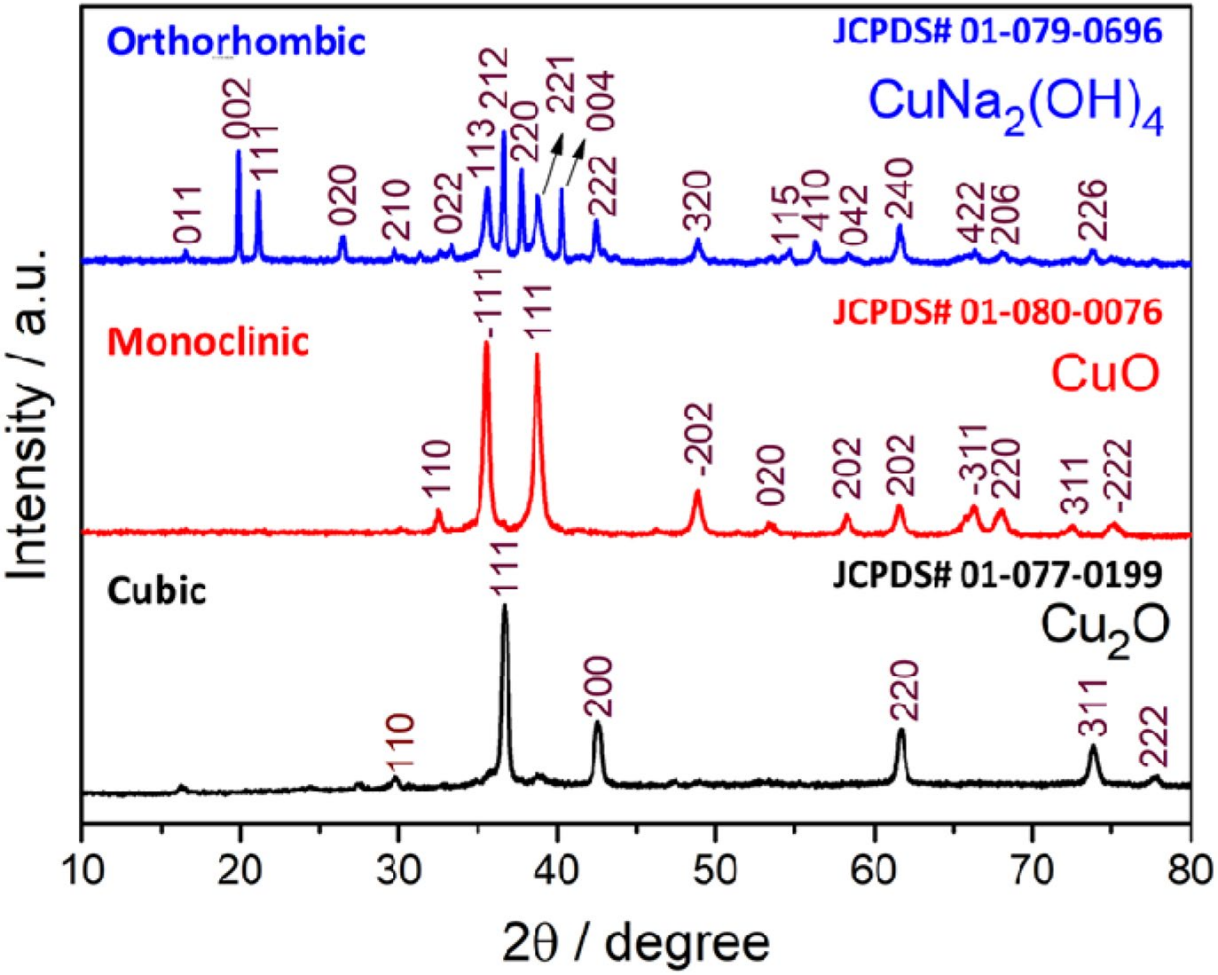
X-ray diffraction pattern of three different phase of copper oxides.

X-ray diffraction is the most widely recognized study to evaluate structural and quality of the samples. Figure 1 shows XRD pattern of different crystalline structures of copper-based metal oxide compounds. The dramatic structural changes obtained by controlling the concentrations of NaOH (0.25, 0.5, 1.0 M) with CuSCN (0.5 M) precursor. For the first time, copper-based three different crystal structures such as cubic (Cu_2_O), monoclinic (CuO) and orthorhombic [CuNa_2_(OH)_4_] were prepared by increasing the concentration of NaOH from 0.25, 0.5 and 1.0 M, respectively. In the XRD pattern of Cu_2_O, all the diffraction peaks can be confirmed to be the cubic structure of Cu_2_O (JCPDS No. 01-077-0199) as displayed in Fig. 1.

The size of powder samples can be estimated reliably from the broadening of diffraction peaks. Additionally, the crystallite size which depends on the width of diffraction peaks was approximately evaluated using the Debye–Scherrer's equation^35^. As can be seen in Fig. 1, all the diffraction peaks obtained from Cu_2_O sample are well matched with standard data (JCPDS No. 01-077-0199). The calculated crystallite size was about 22.2 nm. The diffraction peaks obtained for CuO nanoparticles are well indexed to the monoclinic structure of CuO (JCPDS No. 01-080-0076) with superior crystal quality. No additional peaks were obtained, which demonstrates the high quality of product. The above result clearly shows that 0.5 M NaOH is more favorable concentration for the preparation of high quality monoclinic CuO nanoparticles. The average crystallite size was found to be in the range of 21.6 nm. More interestingly, orthorhombic structure of copper sodium hydroxide (CuNa_2_(OH)_4_) obtained for high concentration of NaOH (1 M) shows all the characteristic diffraction peaks. The strong peaks are well matched with the JCPDS no. 01-079-0696. The average crystallite size of CuNa_2_(OH)_4_ was calculated as 24.7 nm.

**Figure 2:**
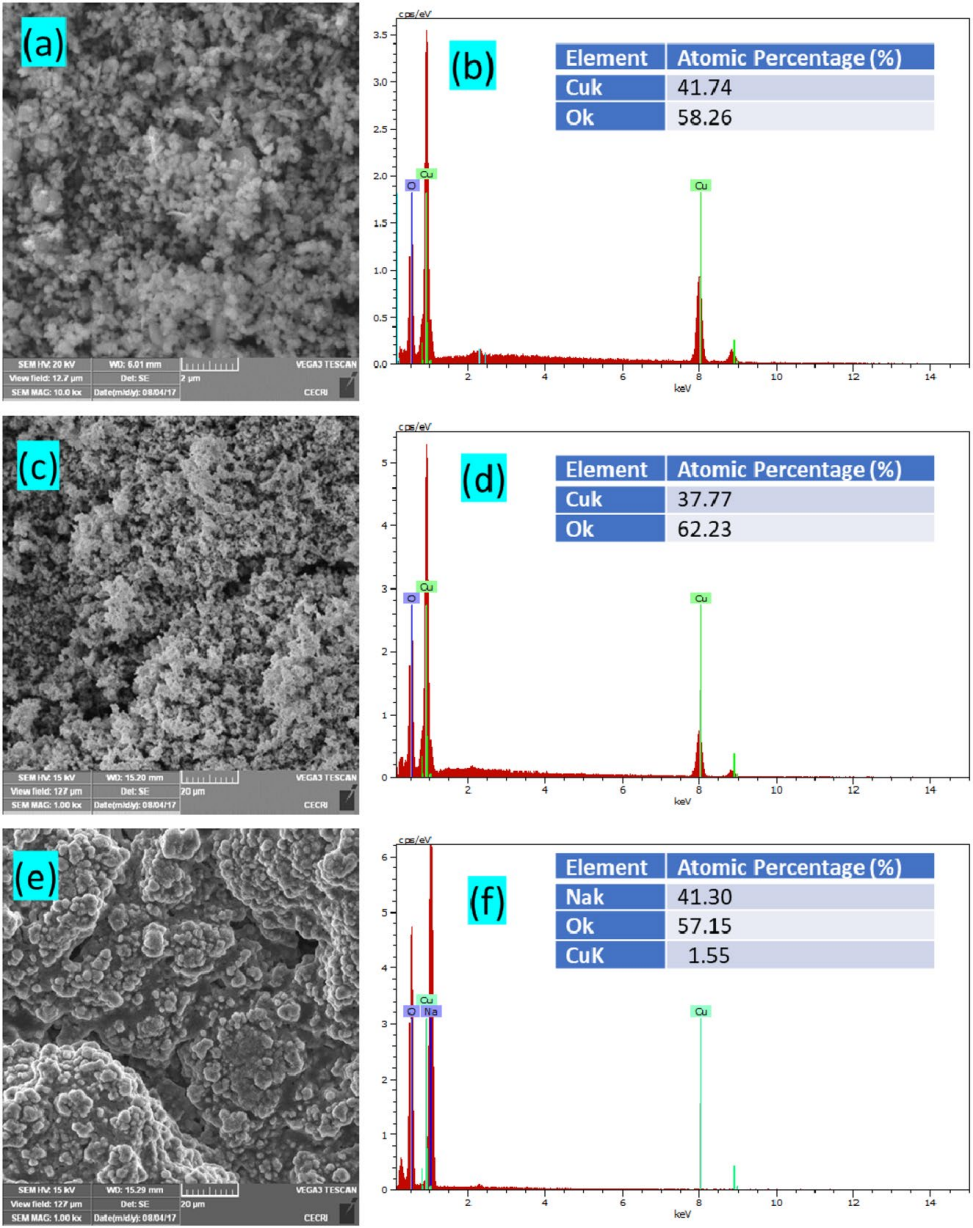
(**a**,**c**,**e**) SEM images and (**b**,**d**,**f**) EDS spectra of Cu_2_O, CuO and CuNa_2_(OH)_4_ nanoparticles, respectively.

The microstructural information of nanostructures was investigated using SEM and TEM micrographs. Figure 2a,c,e shows SEM images of Cu_2_O, CuO and CuNa_2_(OH)_4_ nanoparticles and the corresponding EDAX results are presented in Fig. 2b,d,f, respectively. The SEM micrographs clearly showed that the particles were roughly agglomerated with homogeneous morphologies. The EDAX result showed only the elements present in the spectra for the corresponding samples and not for any other impurities or secondary products. The obtained EDX results confirmed that only Cu and O ions are present in the prepared nanoparticles (Fig. 2b,d) with the same ratio proportion as defined at the time of experiment. The atomic percentage of element of the corresponding samples is presented in table (inset of Fig. 2b,d,e).

**Figure 3:**
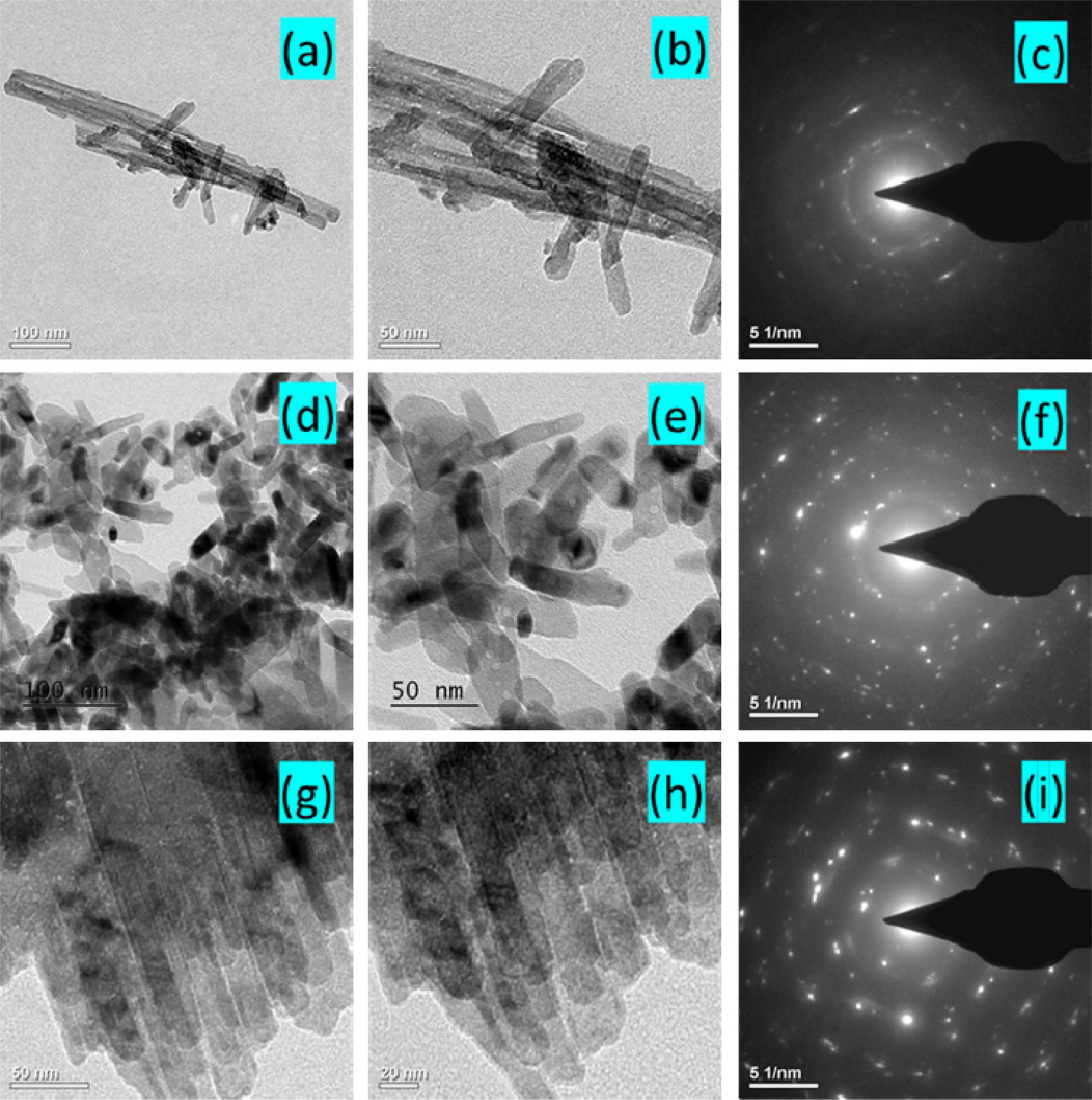
TEM images and SAED patterns of nanoparticles: (**a**–**c**) Cu_2_O nanorods; (**d**–**f**) CuO nano rice and (**g**–**i**) well-aligned CuNa_2_(OH)_4_ nanorods.

To evaluate exact sizes and morphology of the nanoparticles, TEM measurement was performed. Figure 3a,b illustrates the morphology of highly crystalline Cu_2_O nanoparticles. The TEM images showed the presence of rod like morphology with an average diameter of 20–25 nm and length of ~ 500 nm. Uniform beads like morphology of CuO are depicted in Fig. 3d,e. The average size of the nano beads is about 25 nm. As shown in Fig. 3g,h, a bunch of transparent uniform nanorods structure obtained for CuNa_2_(OH)_4_ when 1 M of NaOH was used. TEM results clearly revealed that variation of precursor concentration (NaOH) is not only modified the crystal structure but also tuned the morphology. To further investigate structural information, SAED patterns were recorded for all the three samples of Cu_2_O, CuO and CuNa_2_(OH)_4_, the obtained results are presented in Fig. 3c,f,i, respectively. The well-distinguished fringes in the SAED pattern confirm the highly crystalline nature of samples.

**Figure 4:**
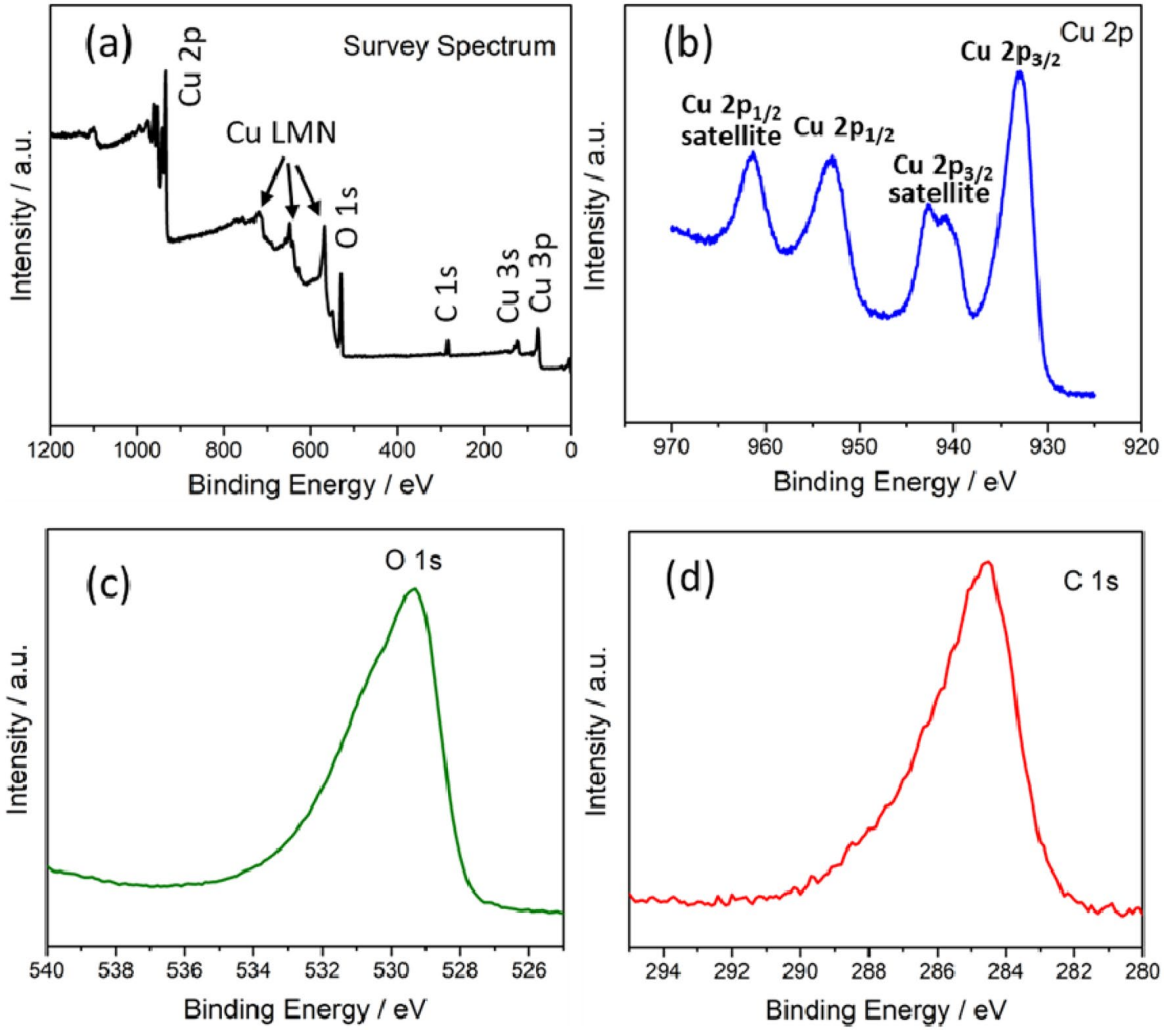
(**a**–**d**) XPS data of CuO nanoparticles: (**a**) Survey spectrum; (**b**) Cu 2p; (**c**) O 1 s and (**d**) C 1 s.

Figure 4a shows survey spectrum of XPS analysis for CuO nanoparticles. The XPS result shows (Fig. 4b) existence of two binding energies, for Cu 2p of CuO sample, at 933.8 eV (Cu 2p_3/2_) and 953.5 eV (Cu 2p_1/2_) with a difference of 19.7 eV, which proves the formation of copper (II) oxide^35^. The presence of two satellite peaks at higher binding energies of 941.4 eV and 961.6 eV are typical of materials having d9 configuration in their ground state that obviously shows the presence of Cu^2+^^35,36^. As well-documented, the spectra of the O1s (Fig. 4c) core level for CuO can be deconvoluted into two components located at 530.10 eV and 530.96 eV^37^. These two parts are attributed to the different chemical state of oxygen, where the peak at lower binding energy ascribed to the oxygen (O^2−^) associating with Cu^2+^ ion in the CuO structure. Figure 4d shows the characteristic binding energy of 283.6 eV corresponding to C 1 s.

**Figure 5:**
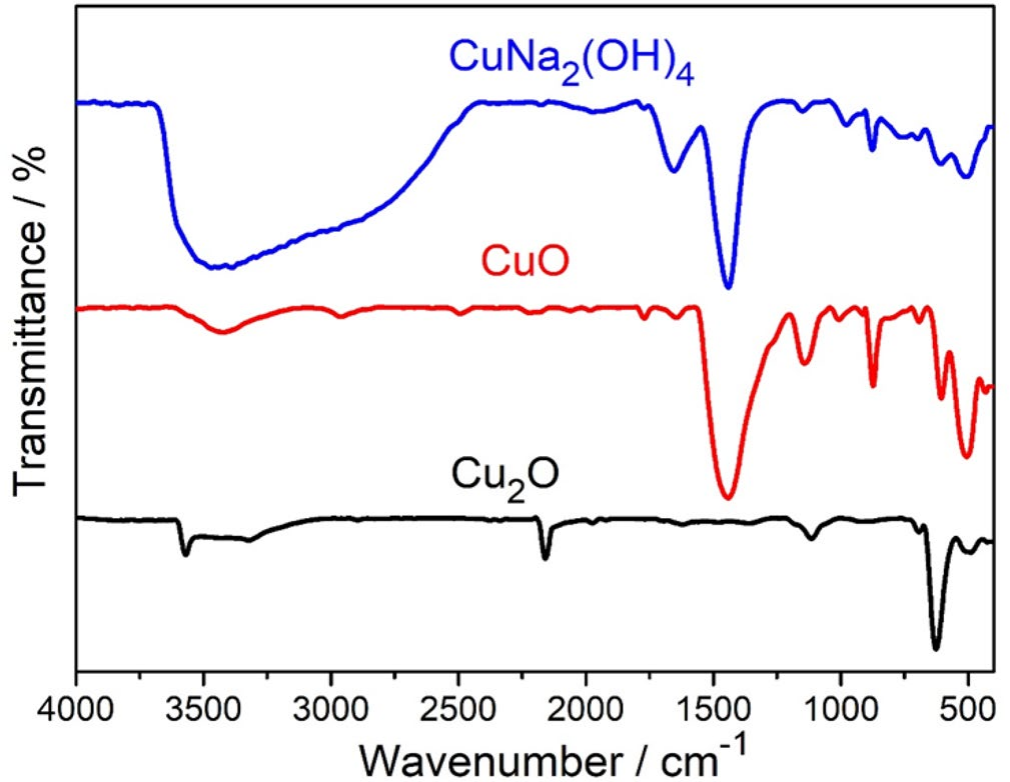
FT-IR spectra of Cu_2_O, CuO and CuNa_2_(OH)_4_ nanoparticles.

FT-IR spectroscopy was used to identify the functional groups present in the materials. Figure 5 shows FT-IR spectra of Cu_2_O, CuO and CuNa_2_(OH)_4_ nanostructures. Among the FT-IR spectra, CuNa_2_(OH)_4_ nanoparticle shows a strong broad absorption band from 2500 to 3750 cm^−1^ corresponds to hydroxyl (OH) functional groups presented in the compound. In the range between 1700 and 1000 cm^−1^ several peaks were observed. The peak around 1611.2 cm^−1^ can be assigned to C=C. The strong peak appeared around 1350 cm^−1^ is attributed to the deformation vibration of C–H band while low intensity peaks appeared between 900 and 700 cm^−1^ also assigned to the aromatic bending vibration of C–H group. The strong absorption peaks observed in the range of 500–700 cm^−1^ are due to the vibrational modes of CuO and Cu_2_O nanostructures^38^.

**Figure 6:**
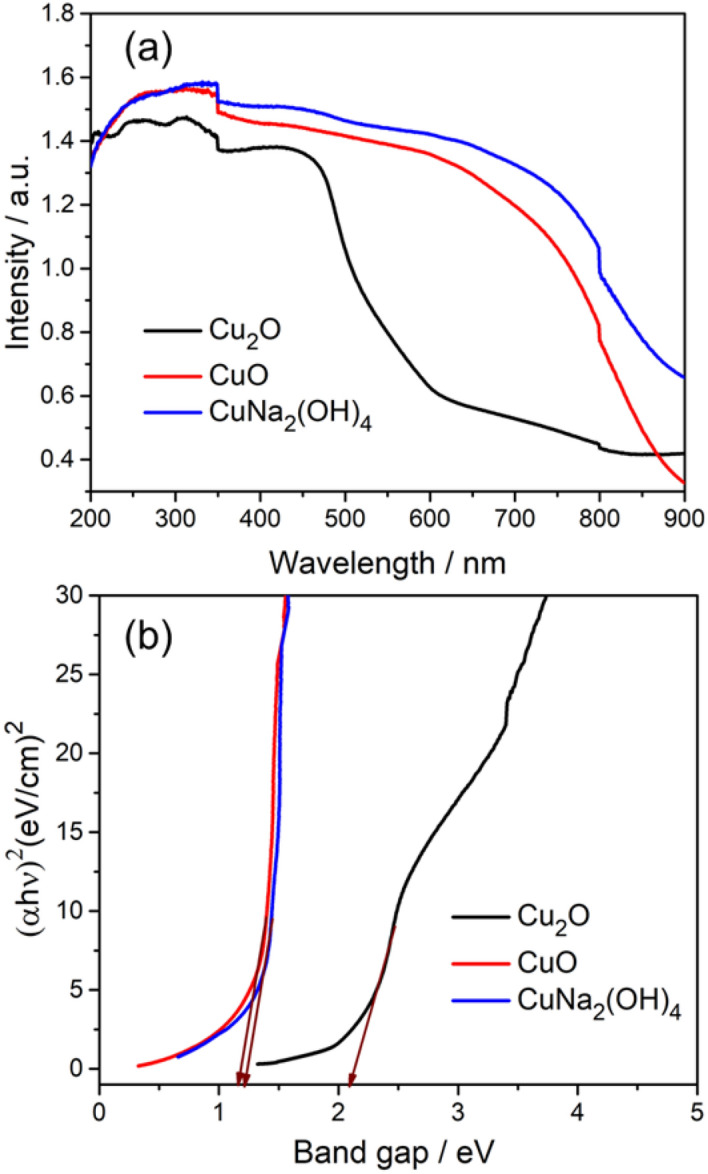
(**a**–**b**) UV absorption and energy band gap of Cu_2_O, CuO and CuNa_2_(OH)_4_ nanoparticles.

Optical absorption behaviour is one of the most important fundamental properties in revealing the energy band gap and optoelectronic applications. Figure 6a,b shows UV–visible absorbance spectra of the as-prepared Cu_2_O, CuO and CuNa_2_(OH)_4_ nanostructures recorded by ultrasonically dispersing in de-ionized water. The absorption peak edges of Cu_2_O, CuO and CuNa_2_(OH)_4_ nanoparticles are observed at 580, 870 and 860 nm, respectively. The broad absorption indicates reduced band gap values and which can boost the conductivity. The band gap of the nanoparticles was determined from absorption values using the Tauc plot^39,40^. The calculated band gap values of the nanoparticles are 2.14, 1.31 and 1.35 eV for Cu_2_O, CuO and CuNa_2_(OH)_4_, respectively.

### Electrochemical studies

#### Comparison of electrocatalytic activity of Cu_2_O, CuNa_2_(OH)_4_ and CuO modified electrodes and pH effect

**Figure 7:**
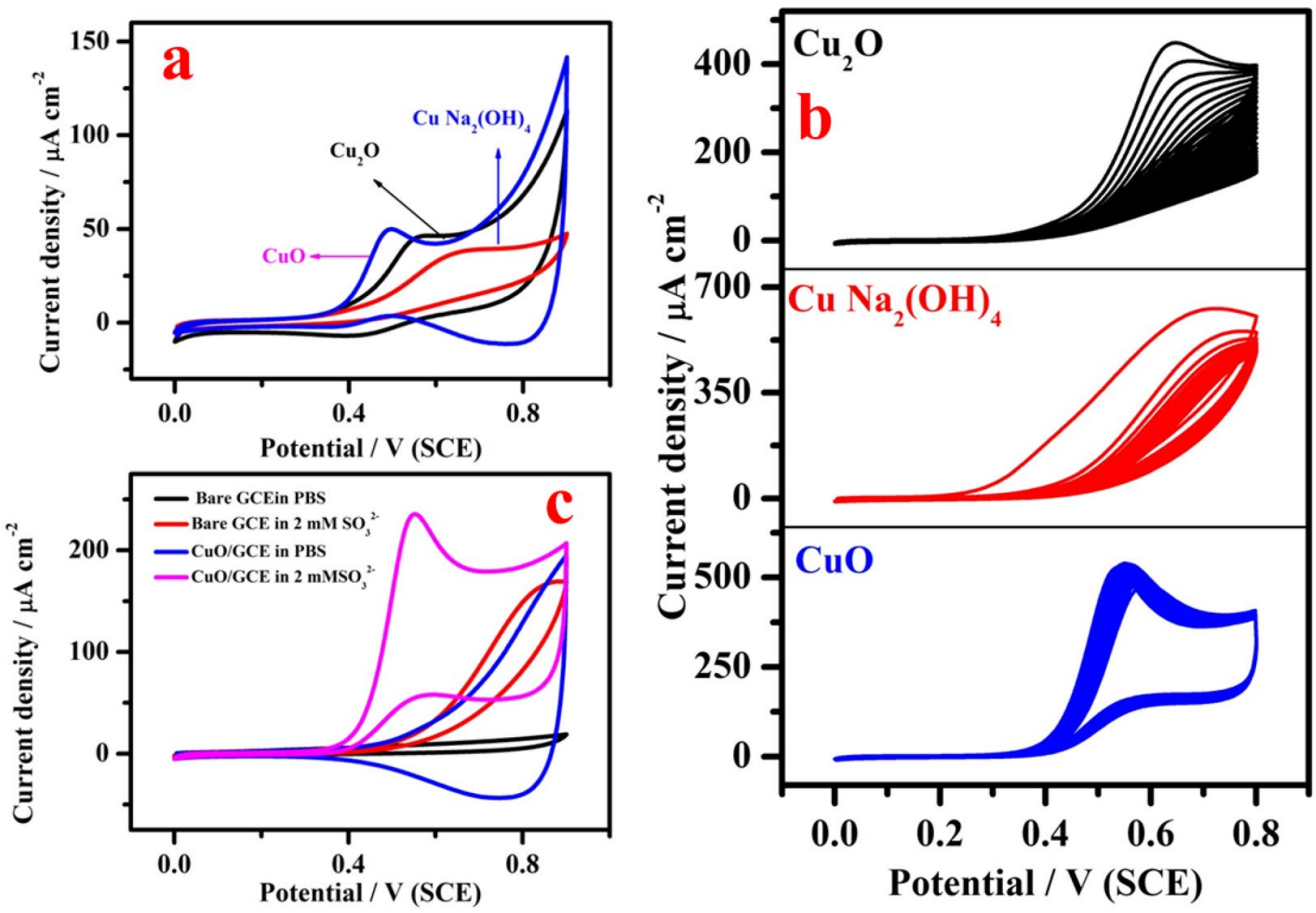
(**a**) CV of 2 mM of SO_3_^2−^ in 0.1 M PBS (pH 7) on CuNa_2_(OH)_4_ (blue colour), Cu_2_O (black colour) and CuO (blue colour) modified electrodes; (**b**) stability studies of Cu_2_O, CuNa_2_(OH)_4_ and CuO modified electrodes using cyclic voltammetry in 5 mM of SO_3_^2−^ and (**c**) CVs of bare GCE (black colour) and CuO modified GCE (blue colour) in pure 0.1 M PBS; bare GCE (red colour) and (pink colour) and CuO/GCE containing 2 mM of SO_3_^2−^. Scan rate = 10 mV s^−1^.

Figure 7a shows CV studies of copper-based metal oxides such as Cu_2_O, CuNa_2_(OH)_4_ and CuO modified electrodes in 0.5 mM SO_3_^2−^ at a scan rate of 5 mV s^−1^. All the three catalysts, CuNa_2_(OH)_4_, Cu_2_O and CuO, show electrocatalytic response towards SO_3_^2−^ oxidation at the potential of 640, 550 and 490 mV, respectively. The potential difference between CuNa_2_(OH)_4_–Cu_2_O, Cu_2_O–CuO and CuNa_2_(OH)_4_–CuO were 90 mV, 150 and 60 mV, respectively. The current value was found to be higher for CuO compared to CuNa_2_(OH)_4_ and Cu_2_O. It shows that the CuO has high conductivity with attractive electrocatalytic ability towards oxidation of SO_3_^2−^. As shown in Fig. 7b, electrochemical stability studies were also carried out for all the three materials in the presence of 5 mM SO_3_^2−^ using cyclic voltammetry by continuously recording 30 cycles. All the three modified electrodes show the electrochemical response for the oxidation of SO_3_^2−^. Unfortunately, the current response decreases continuously after few cycles in the case of Cu_2_O and CuNa_2_(OH)_4_. On the other hand, CuO shows stable response and the current decrease is negligible in comparison with other two modified electrodes. The above cyclic voltammetry studies clearly demonstrated that CuO possesses higher catalytic activity and better stability compared to other two electrocatalysts. Therefore, CuO modified electrode was chosen as the best catalyst and hence most favourable for further studies on electrochemical sensing of SO_3_^2−^. As shown in Fig. 7c, electrooxidation of SO_3_^2−^ occurs on CuO/GCE at less positive potential with enormous current compared to the same on bare GCE at around 0.8 V with lower current value and featureless voltammogram. The simple mechanism for the electrochemical oxidation of SO_3_^2−^ by CuO/GCE is given below.

1 $${\text{SO}}_{{3}}^{{{2} - }} + {\text{H}}_{{2}} {\text{O}} \to {\text{SO}}_{{4}}^{{{2} - }} + {\text{2H}}^{ + } + {\text{2e}}^{ - }$$

We anticipated that the electrochemical oxidation of SO_3_^2−^ would be depend on the solution pH. In order to examine the DPV response of SO_3_^2−^ at CuO/GCE, measurement was carried out in 3 mM SO_3_^2−^ with varying pH of 6.0, 6.5, 7.0, 7.5 and 8.0 as illustrated in Fig. S1a. A plot of current density versus pH is shown in Fig. S1b. It can be noticed that the oxidation peak current was higher in the case of pH 7. Further increase in pH leads to decrease in the oxidation current. This phenomenon clearly explains that proton is involved in the process of electrochemical oxidation of SO_3_^2−^. Hence, 0.1 M PBS (pH 7) was selected as supporting electrolyte throughout the experiments.

#### Scan rate effect

**Figure 8:**
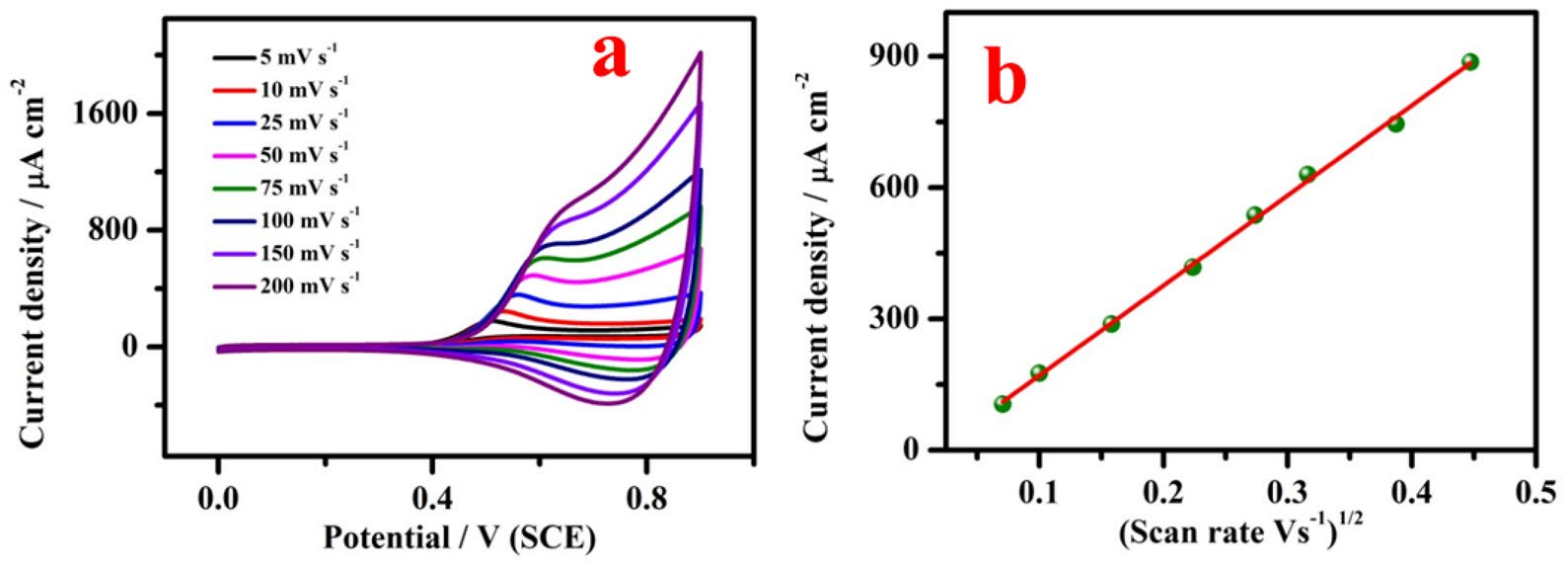
(**a**) Effect of scan rate on the CV of CuO/GCE in 2 mM of SO_3_^2−^ at varying the scan rate from 5, 10, 25, 50, 75, 100, 150 and 200 mV s^-1^ and (**b**) corresponding plot of peak current versus square root of scan rate.

Figure 8a shows CVs of CuO modified electrode at different scan rate of 5, 10, 25, 50, 75, 100, 150 and 200 mV s^−1^ in 1 mM of SO_3_^2−^. CV result clearly exhibited that the oxidation peak current increases with raising the scan rate. While increasing the scan rate, the potential shifted towards higher potential value. Figure 8b shows a plot of SO_3_^2−^ oxidation peak current density (Ipa) versus square root of the scan rate from 5 to 200 mV s^−1^. Further, log (current density) vs log (scan rate) plot exhibits a slope value of ~ 0.5 (not shown here). The above results show that the overall reaction was controlled by diffusion^41^.

#### Concentration effect

**Figure 9:**
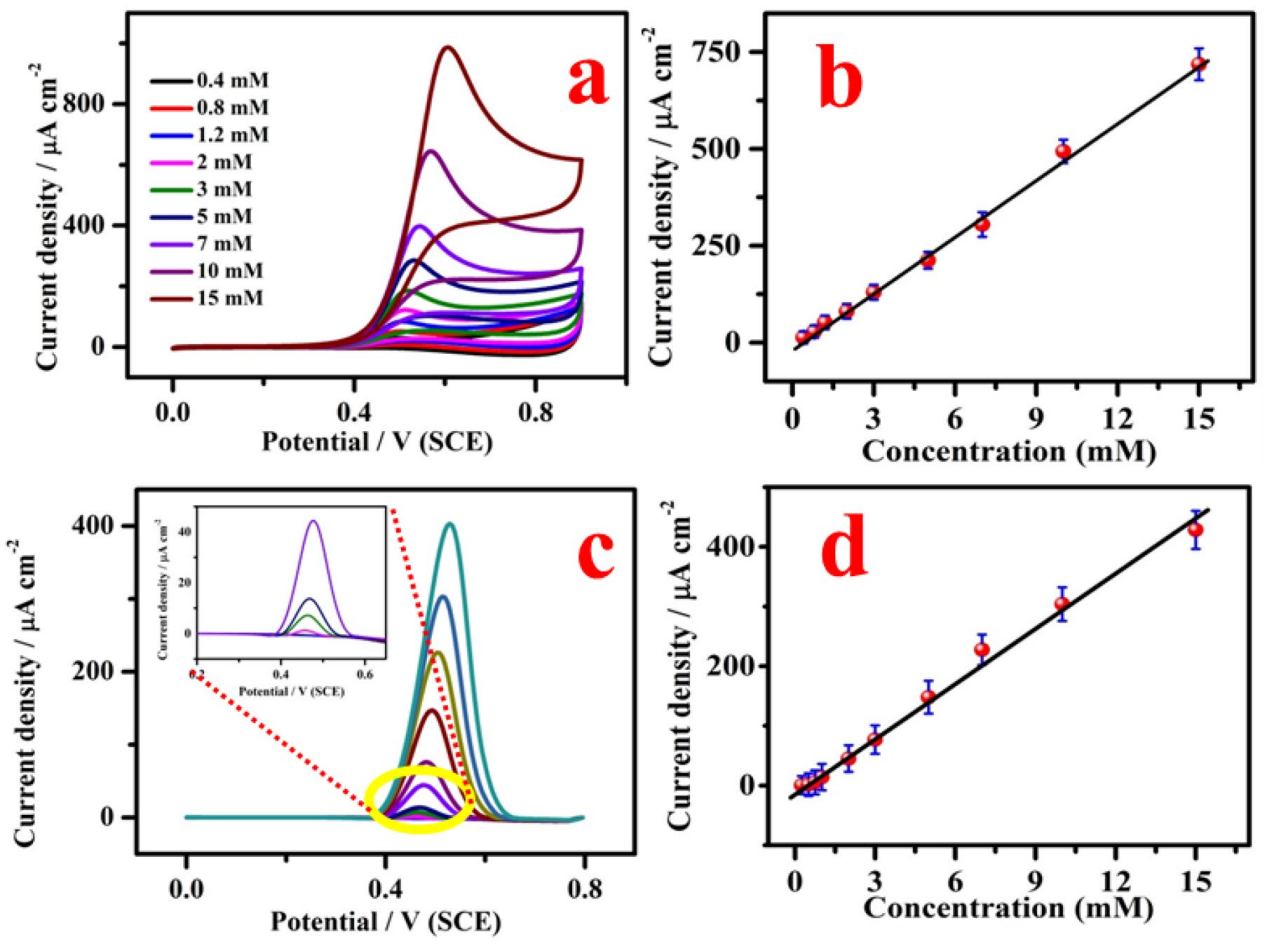
(**a**) CV of CuO/GCE in 0.1 M PBS (pH 7.0) containing 0.4, 0.8, 1.2, 2.0, 3.0, 5.0, 7.0, 10.0 and 15.0 mM of SO_3_^2−^; (**b**) corresponding calibration curve (n = 3). (**c**) Differential pulse voltammogram of CuO modified electrode in 0.1 M PBS pH 7 concentration ranges from 0.2, 0.3, 0.5, 0.75, 1.0, 2.0, 3.0, 5.0, 7.0, 10.0 and 15.0 mM of SO_3_^2−^. Insert shows the low concentration region and (**d**) a plot of current density with respect to the concentration (n = 3). (DPV parameters: step potential = 0.0050 V; modulation amplitude = 0.0250 V; modulation time = 0.20 s and interval time-0.5 s).

Figure 9a demonstrates the electrocatalytic response of CuO/GCE for the detection of different concentration (0.4, 0.8, 1.2, 2.0, 3.0, 5.0, 7.0, 10.0 and 15.0 mM) of SO_3_^2−^. It clearly showed that CuO/GCE has appreciable electrocatalytic activity for the sensing of SO_3_^2−^. From the Fig. 9b, the analytical parameter such as sensitivity, limit of detection (LOD) were found to be 48.5 µA cm^−2^ mM^−1^ and 2.23 µM (LOD = 3σ/S; σ is the standard deviation and S is the sensitivity) with the correlation coefficient (R^2^) of 0.9953. The corresponding linear regression equation was y = 48.5C (SO_3_^2−^) + (− 14.7). Similarly, the performance of CuO/GCE for the detection of SO_3_^2−^ was explored by DPV technique also. As shown in Fig. 9c, SO_3_^2−^ oxidation peak was observed at 0.47 V in DPV technique. The oxidation current increases with increasing SO_3_^2−^ concentration from 0.005 to 15 mM. From the calibration curve shown in Fig. 9d, the linear range, LOD and sensitivity were found to be 0.005–15 mM, 1.42 µM and 29.93 µA cm^−2^ mM^−1^ (R^2^ = 0.9906), respectively. The linear regression equation was y = 29.93C (SO_3_^2−^) + (− 8.9). Table 1 displays the electrochemical sensor parameter of our proposed sensor along with previous reports based on metal, metal oxides and carbon materials. It can be noticed that the performance of CuO/GCE based sensor is comparable with previous works.

**Table 1:** Displays the electrochemical sensor parameter of different electrochemical sensors for the determination of SO_3_^2−^.

No.	Type of modified electrodes	Supporting electrolyte	Linear range	Sensitivity	Limit of detection	References
1	^a^CoHCF/CPE	0.1 M PBS	1.00–7.83 mM	4.61 µA/mM	2.87 µM	^17^
2	Copper-salen	0.5 M KCl	4.0–69 µM	–	1.2 µM	^18^
3	CuO-NS	0.1 M PBS	50–1600 µM	4.02 µA cm^−2^ mM^−1^	21.10 µM	^22^
4	p-CoP-II film	0.1 M NaNO_3_	1–10 mM	19.62 µA/mM	0.195 mM	^42^
5	CuHCF/CNT/CPE	0.1 M KNO_3_	0.5–50 mg l^−1^	0.17 µA mM^−1^	5.0 µM	^43^
6	^b^PANI/CuHCF/GC	0.1 M KCl–HCl	0.0043–0.39 mM	0.0624 µA mM^−1^	0.6 µM	^44^
7	NiO-Nanoplatlet	0.1 M PBS	16.2 µM–0.6 mM	2.8 µA cm^−2^ mM	8.8 µM	^45^
8	^c^CuCoHCF/CPE	0.15 M PBS	0.005–5.0 mM	–	1 µM	^46^
9	2,7-BFEFMCPE	0.1 M PBS	4 µM–20 mM	–	3.0 µM	^47^
10	CHIT-Fc/MWCNTs/GCE	0.1 M PBS	5 µM–1.5 mM	13.08 µA mM^−1^	2.8 µM	^48^
11	NiPCNF/CCEs	–	2 µM–2.0 mM	13.05 nA mM^−1^	0.5 µM	^49^
12	CuO/GCE	0.1 M PBS	5 µM–15 mM	29.93 µA cm^−2^ mM^−1^	1.42 µM	This work

^a^Cobalt hexacyanoferrate.

^b^Polyaniline-coated copper hexacyanoferrate.

^c^Copper-cobalt hexacyanoferrate.

#### Real sample analysis

For the practical application, CuO/GCE was used to analyze wine samples, which were purchased from local wine shop and used as real sample to detect the presence of SO_3_^2−^. Using PBS solution, the wine samples were diluted and the required amount of the sample was injected into the supporting electrolyte without further pre-treatment. The CuO/GCE was used for sensing SO_3_^2−^ in the wine samples. A characteristic catalytic peak of SO_3_^2−^ was observed at 0.5 V. Cyclic voltammetric measurement was performed for the estimation of SO_3_^2−^ in the wine sample as shown in Fig. S2a and the corresponding calibration curve is displayed in Fig. S2b. The sensor parameters of real samples obtained are summarized in Table 2. The proposed sensor shows recovery in the range of 99–100.9%, which indicates that the proposed CuO based electrochemical sensing platform can be used for the determination of SO_3_^2−^ in wine samples.

**Table 2:** Real sample analysis of SO_3_^2−^ trace in wine samples by DPV technique using CuO/GCE.

Analysed product	SO_3_^2−^ concentration (mg/100 ml) wine sample	SO_3_^2−^ concentration after addition of 100 mg/100 ml wine solution	Degree of recovery (%)	RSD (%)
Wine sample 1	37.3	136.4	100.9	3.2
Wine Sample 2	34.1	135.1	99.0	4.1

#### Repeatability, stability, fabrication reproducibility and interference studies of CuO/GCE

**Figure 10:**
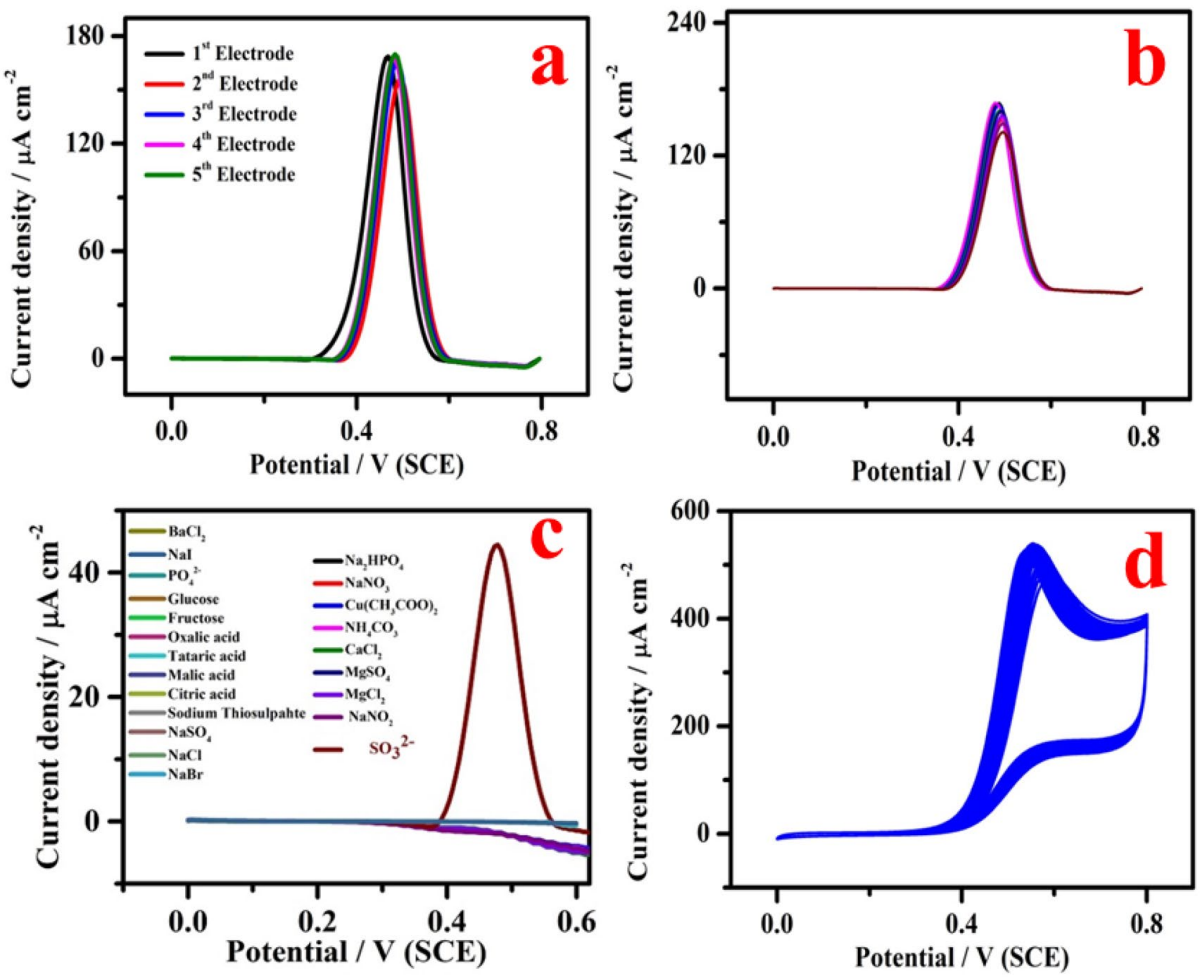
(**a**, **b**) Fabrication reproducibility (five electrodes) and repeatability of CuO/GCE by DPV technique in the existence of 5 mM SO_3_^2−^. (**c**) Selectivity of CuO/GCE by DPV response in the presence of 0.5 mM SO_3_^2−^ and (**d**) stability study of CuO/GCE for the electrooxidation of 5 mM SO_3_^2−^. (DPV parameters: step potential = 0.0050 V; modulation amplitude = 0.0250 V; modulation time = 0.20 s and interval time-0.5 s).

In order to assess the fabrication reproducibility of proposed sensor, five modified electrodes were fabricated under identical fabrication condition then employed to record the electrochemical response of 5 mM SO_3_^2−^ and the obtained results are displayed in Fig. 10a. The RSD was found to be 4.1%, which indicates good reproducibility of the sensors made in the same way. Typically, the response of a modified electrode to an analyte decreases after several measurements. In this context, we have evaluated the repeatability of CuO modified electrode in sulphite estimation by continuously monitoring the electrochemical response of 5 mM SO_3_^2−^ for every 4 min interval for 10 measurements using DPV techniques as shown in Fig. 10b. The RSD for the obtained currents was 3.6%, which indicates extraordinary repeatability of the sensor in SO_3_^2−^ determination. In order to examine the selectivity of CuO/GCE for the detection of SO_3_^2−^, the influence of foreign species on the detection of 0.5 mM of SO_3_^2−^ was investigated by DPV. The study was carried out under 200 fold higher concentration of interfering species including BaCl_2_, NaI, PO_4_^2−^, glucose, fructose, oxalic acid, tartaric acid, malic acid, citric acid, sodium thiosulphate, NaCl, NaBr, Na_2_HPO_4_, NaNO_3_, Cu(CH_3_COO)_2_, (NH_4_)_2_CO_3_, CaCl_2_, MgCl_2_ and Na_2_SO_4_ which are not influencing the sensing of SO_3_^2−^ as shown in Fig. 10c. Further, CV technique was used for the measurement of stability of the proposed sensor by continuously recording 50 cycles in 5 mM SO_3_^2−^, as displayed in Fig. 10d, the decrease in current response was negligible. The above studies reveal that the proposed senor demonstrates good repeatability, selectivity, fabrication reproducibility and stability for the sensing of SO_3_^2−^.

**Figure 11:**
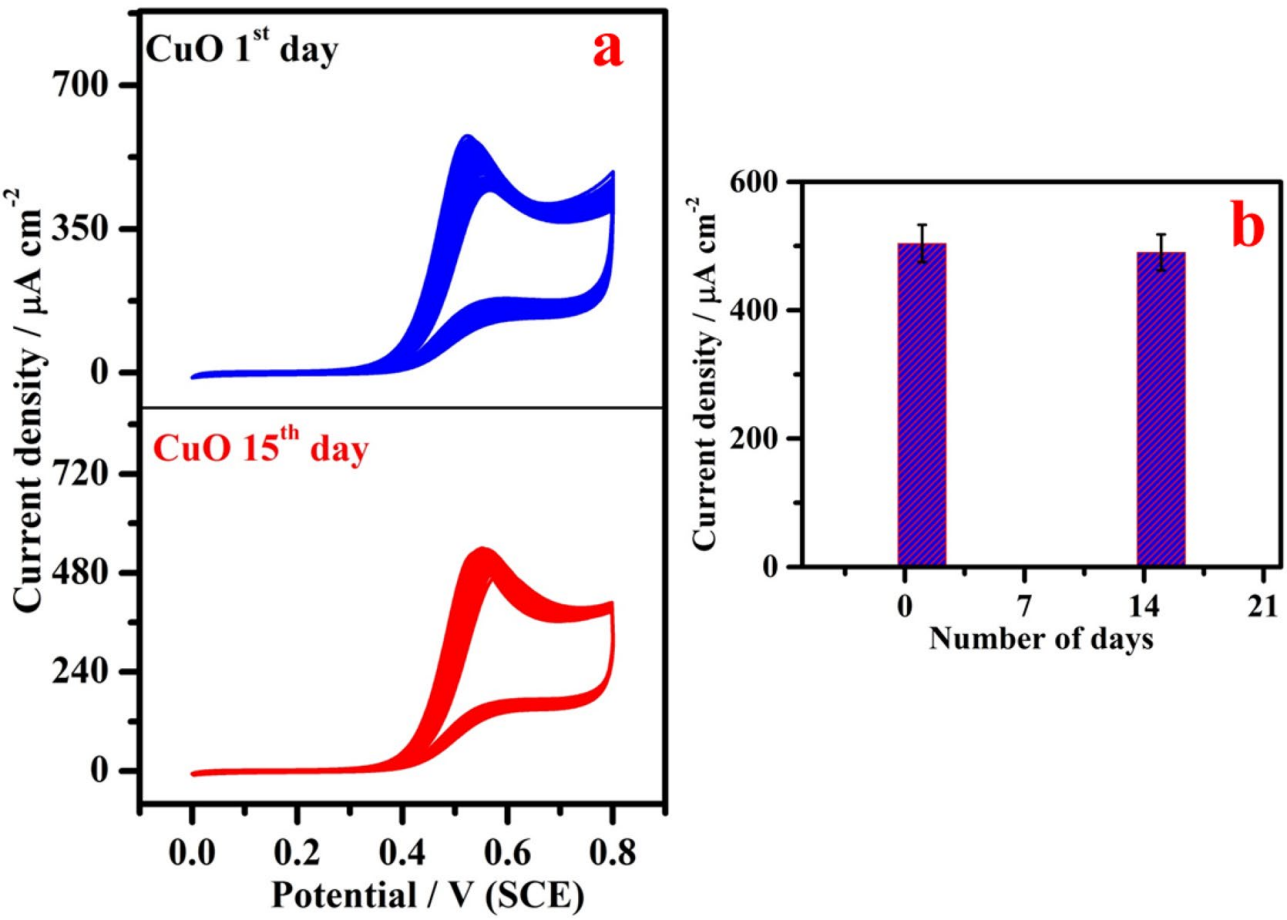
(**a**) Long-time stability of CuO/GCE for the electrooxidation of 5 mM SO_3_^2−^: cyclic voltammetric response on 1st day and 15th day and (**b**) Corresponding flow chat (current density *vs* number of days).

The long-time stability of the CuO/GCE was examined by CV as illustrated in Fig. 11a for the detection of 5 mM SO_3_^2−^. After 15 days, the experiment was carried out using the same procedure and the electrode retained 94% current value, which indicates high storage stability of CuO based sensor in SO_3_^2−^ estimation and the proposed electrode was stored in air atmosphere when not in use. Corresponding flow chat was displayed in Fig. 11b.

## Conclusions

The Cu_2_O, CuNa_2_(OH)_4_ and CuO nanostructures have been effectively synthesized by single stage chemical method. The microscopic studies obviously revealed the phase purity, surface morphology, functional groups and elemental composition. Electrocatalytic activity was evaluated using CV and DPV techniques. The Cu_2_O, CuNa_2_(OH)_4_ and CuO catalysts exhibited electrocatalytic activity, however, higher current was observed in the case of CuO/GCE for the detection of SO_3_^2−^. The limit of detection and sensitivity of CuO/GCE for the electrochemical estimation of SO_3_^2−^ were found to be 1.42 µM and 29.93 µA cm^−2^ mM^−1^, respectively. The low-cost and environmental friendly CuO modified electrode is an excellent platform for the electrooxidation of SO_3_^2−^ as well as exhibited appreciable electrochemical durability in neutral medium. The present work proposes a new methodology for the sensing of SO_3_^2−^ using CuO sensor. This proposed sensor was used for the quantitative detection of SO_3_^2−^ in commercial wine samples thereby opening a new avenue for assessing food quality.

## Supplementary Information

Supplementary Information.
