# Supplementary material for: Structural and morphological tuning of Cu-based metal oxide nanoparticles by a facile chemical method and highly electrochemical sensing of sulphite
Source: Sci Rep. 2021 Feb 9;11:3413. doi: 10.1038/s41598-021-82741-z (PMC7873194; doi:10.1038/s41598-021-82741-z)
Supplement: Supplementary file 1 — Supplementary Information. [file 41598_2021_82741_MOESM1_ESM.docx]

**Structural and morphological tuning of Cu-based metal oxide nanoparticles by a facile chemical method and highly electrochemical sensing of sulphite**

Velayutham Sudha,^a,b^ Govindhasamy Murugadoss,^a,c*^ Rangasamy Thangamuthu,^a,b^

*^a^Electroorganic and Materials Electrochemistry (EME) Division, CSIR-Central Electrochemical Research Institute (CSIR-CECRI), Karaikudi-630 003, Tamil Nadu, India*

*^b^*Academy of Scientific and Innovative Research (AcSIR), Ghaziabad – 201 002, India

*^c^Centre for Nanoscience and Nanotechnology, Sathyabama Institute of Science and Technology, Chennai-600 119, Tamil Nadu, India.*

**pH effect:**


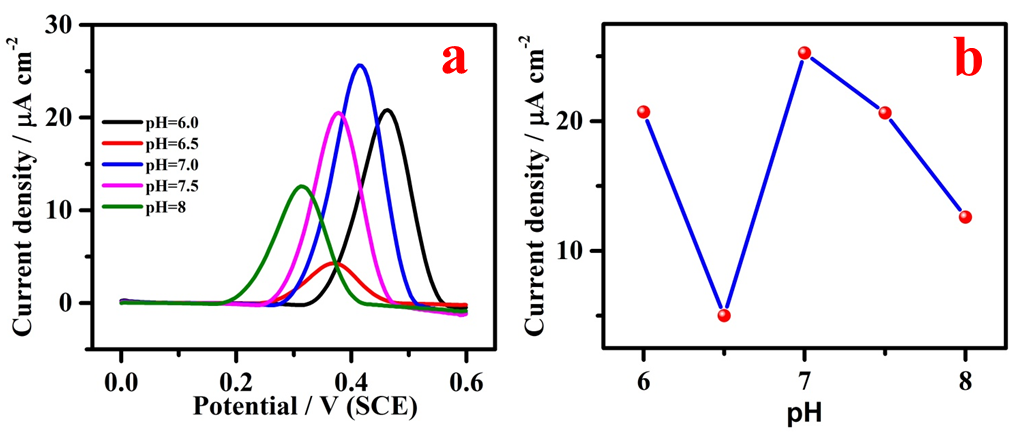


**Figure S1 (a)** DPV of CuO/GCE for 3 mM of SO_3_^2-^ oxidation and (b) Corresponding current vs pH plot. (DPV parameters: Step potential=0.0050 V; Modulation amplitude=0.0250 V; Modulation time=0.20 sec and Interval time-0.5 sec).


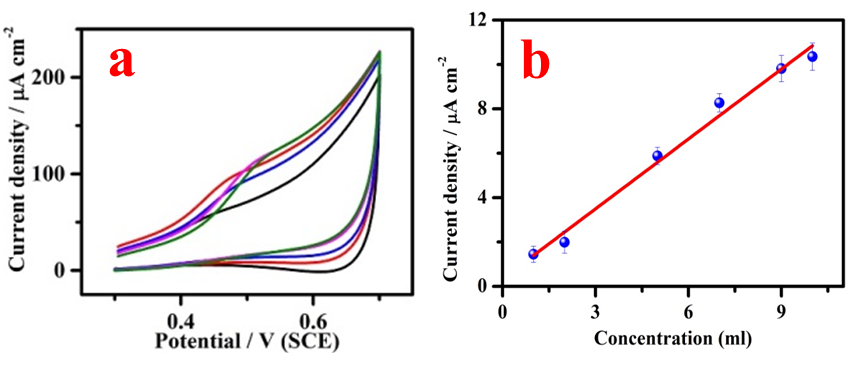


**Figure S 2** (a) CV of CuO/GCE in 0.1 M PBS (pH=7.0) in the presence of 1.0, 2.0, 5.0, 7.0, 9.0 and 10 ml of wine sample and (b) Corresponding calibration curve.
